# Risk Factors Associated with Sleep Disturbance following Traumatic Brain Injury: Clinical Findings and Questionnaire Based Study

**DOI:** 10.1371/journal.pone.0076087

**Published:** 2013-10-01

**Authors:** Lijun Hou, Xi Han, Ping Sheng, Wusong Tong, Zhiqiang Li, Dayuan Xu, Mingkun Yu, Liuqing Huang, Zhongxin Zhao, Yicheng Lu, Yan Dong

**Affiliations:** 1 Department of Neurosurgery, Shanghai Institute of Neurosurgery, PLA Institute of Neurosurgery, Changzhang Hospital, Second Military Medical University, Shanghai, China; 2 Department of Neurosurgery, Huashan Hospital, Fudan University, Shanghai, China; 3 Department of Neurosurgery, Pudong New Area People’s Hospital, Shanghai, China; 4 Department of Neurosurgery, Fengxian Central Hospital, Shanghai, China; 5 Department of Neurology, Changzhang Hospital, Second Military Medical University, Shanghai, China; Hôpital du Sacré-Coeur de Montréal, Canada

## Abstract

**Background:**

Sleep disturbance is very common following traumatic brain injury (TBI), which may initiate or exacerbate a variety of co-morbidities and negatively impact rehabilitative treatments. To date, there are paradoxical reports regarding the associations between inherent characteristics of TBI and sleep disturbance in TBI population. The current study was designed to explore the relationship between the presence of sleep disturbance and characteristics of TBI and identify the factors which are closely related to the presence of sleep disturbance in TBI population.

**Methods:**

98 TBI patients (72 males, mean age ± SD, 47 ± 13 years, range 18-70) were recruited. Severity of TBI was evaluated based on Glasgow Coma Scale (GCS). All participants performed cranial computed tomography and were examined on self-reported sleep quality, anxiety, and depression.

**Results:**

TBI was mild in 69 (70%), moderate in 15 (15%) and severe in 14 (15%) patients. 37 of 98 patients (38%) reported sleep disturbance following TBI. Insomnia was diagnosed in 28 patients (29%) and post-traumatic hypersomnia in 9 patients (9%). In TBI with insomnia group, 5 patients (18%) complained of difficulty falling asleep only, 8 patients (29%) had difficulty maintaining sleep without difficulty in initial sleep and 15 patients (53%) presented both difficulty falling asleep and difficulty maintaining sleep. Risk factors associated with insomnia were headache and/or dizziness and more symptoms of anxiety and depression rather than GCS. In contrast, GCS was independently associated with the presence of hypersomnia following TBI. Furthermore, there was no evidence of an association between locations of brain injury and the presence of sleep disturbance after TBI.

**Conclusion:**

Our data support and contribute to a growing body of evidence which indicates that TBI patients with insomnia are prone to suffer from concomitant headache and/or dizziness, report more symptoms of anxiety and depression and severe TBI patients are likely to experience hypersomnia.

## Introduction

Traumatic brain injury (TBI) constitutes a major health and socioeconomic problem worldwide, which is one of the leading causes of death and disability among children and young adults. TBI can result in a broad range of adverse outcomes that may persist for an extended period of time. Among the persisting symptoms of TBI, disturbance of sleep is very common, varying across studies from 30% to 80% [1-10]. Disrupted sleep may initiate or exacerbate a variety of co-morbidities, such as anxiety, depression, irritability, fatigue, cognitive deficits, pain and functional impairments, and negatively impact rehabilitative treatments [11,12]. Moreover, sleep disruption is also associated with worse quality of life, increased economic costs for the health system, increased risk for hypertension, diabetes and all-cause mortality [13,14].

Some data show that less severe TBI might be associated with greater sleep complaints [3,6,7,11,15], probably because individuals with a milder severity of TBI are prone to struggle to restore their lifestyles and have better insight into the impact of injury. On the contrary, other studies demonstrate that a milder severity of TBI doesn’t contribute significantly to the prediction of the presence of sleep disruption [8,16-19]. Furthermore, only a few data describe whether the location of TBI may be a risk factor for the presence of post-TBI sleep disturbance [14,17]. Currently, a quantity of studies recruits participants from rehabilitation settings rather than hospitals/trauma centers. Some of the clinical characteristics of patients, especially assessment of structural damages by neuroimaging, are often obscure, which impede to identify the association between inherent characteristics of TBI (injuries in specific brain structures, homeostasis misbalance of neurotransmitter systems, hypopituitarism, etc.) and sleep disturbance.

The current study was designed to: investigate the frequency and types of sleep problems following TBI; explore the relationship between the presence of sleep disturbance and characteristics of TBI, such as the severity of TBI and location of TBI; identify the factors which are closely related to the presence of sleep disturbance in TBI population.

## Patients and Methods

### Patients

This study comprised 98 patients (72 males, mean age ± SD, 47 ± 13 years, range 18-70) admitted to Changzheng Hospital, Pudong New Area People’s Hospital and Fengxian Central Hospital immediately after injury between March 2008 and March 2011. There were no obvious differences among three hospitals in the availability and training of clinical staff, ICU structure and commitment to trauma specific training. Criteria for patient selection were: 1) adults, 2) with acute, first-ever TBI, and (3) with positive findings on cranial CT scans. Patients were excluded if they had shift work, suffered sleep and/or psychiatric disorders prior to TBI, or underwent the treatment of sedating medications.

The protocol for the study was approved by the ethics committee of Second Military Medical University, or Pudong New Area People’s Hospital or Fengxian Central Hospital. Patients were deemed to have a capacity/ability to consent if they had the ability to communicate a reasoned choice regarding participation and the ability to understand relevant information about the study, including the purpose of the study, the procedures to which the participants would be exposed, the benefits or risks to participants and the right to decide whether or not to participate in the research study. Informed written consent for study participation was obtained from all patients.

### Follow-up

Telephone assessments were conducted to evaluate clinical outcomes sleep disturbance and psychiatric problems after TBI between August and October 2012. The phone interview consisted of 3 questionnaires including the Pittsburgh Sleep Quality Index (PSQI), Epworth Sleepiness Scale (ESS) and Hospital Anxiety and Depression Scale (HADS), and a structured set of questions about other residual symptoms since TBI including headache and/or dizziness, pain, cognitive disturbance, changes in personality and general behavior, and treatment of sleep disturbance and psychiatric problems. The interviewers were experienced with telephone surveys and received additional specific training on the administration of the structured telephone interview.

### Severity of TBI

The initial evaluation of severity of TBI took place within the first 24 hours after injury, during hospitalization based on Glasgow Coma Scale (GCS). A subject was classified as having a severe TBI if his or her GCS score was less than 9. A subject was classified as having a moderate TBI with a GCS of 9-12 and a mild TBI with a GCS of 13-15. All patients underwent CT scans, which demonstrated relatively precise craniocerebral structural damage. Participants were deemed as loss of consciousness (LOC) according the following criteria: (1) patients were completely or near-completely lack of responsiveness to people and other environmental stimuli in the emergency room or on admission. (2) patients could recall no information about the accident occurring shortly before, during, or immediately after the accident [20].

### Definition of sleep disturbance

Sleep disturbance and psychiatric symptoms were identified using various questionnaires, which were validated in Chinese language [21-23]. Participants completed PSQI to measure sleep quality and ESS to measure daytime sleepiness. Anxiety and depression symptoms were measured using HADS. Insomnia was evaluated and operationalized by International classification of Sleep Disorder 2 (ICSD-2) [24]. Patients had to (1) report insomnia symptoms defined either as (a) a sleep latency of 30 min or more, (b) nocturnal awakenings totaling 30 min or more; (c) a sleep duration of 6 hrs or less despite adequate opportunity and circumstances for sleep; (2) present insomnia symptoms at least 3 nights per week; (3) have a complaint of at least one negative daytime effects (e.g. fatigue, impaired functioning, or mood disturbances) attributed to poor sleep; and (4) have an insomnia duration of at least 1 month. Post-traumatic hypersomnia was defined as increased sleep need per 24 h (at least 2 h more than before TBI).

### Statistical analyses

All participants were separated into 3 groups: TBI without sleep disturbance, TBI with insomnia and TBI with hypersomnia groups. For univariate analyses, the Kruskal-Wallis tests were performed to test whether the three groups originated from the same distribution. When the Kruskal-Wallis test leads to significant results, at least one of the samples is different from the other samples. The test does not identify where the differences occur or how many differences actually occur. Group comparisons were performed with Chi-square tests for categorical and dichotomous data and nonparametric analysis of variance (ANOVA) or analysis of covariance (ANCOVA) tests for continuous data. Multivariate regression analyses were determined using a logistic regression model. Continuous data are expressed as mean±SD. Statistical analyses were performed with SPSS version 19.0. Results were considered significant if associated *p*-values were less than 0.05.

## Results

### Demographic and clinical characteristics of participants

98 patients participated in the present study, including 63 patients from Changzheng Hospital, 19 from Pudong New Area People’s Hospital and 16 from Fengxian Central Hospital. All patients underwent CT scans on admission. The demographic and clinical characteristics of participants were listed in Table 1. The causes of injury in the sample included motor vehicle accident, fall, assault, and others. The predominant cause of injury was motor vehicle accident, accounting for 61 of all cases (63%). TBI was mild in 69 (70%), moderate in 15 (15%) and severe in 14 (15%) patients. Median GCS was 15 (mean ± SD 12.79 ± 3.13, range 4-15). On average, patients were 33 months post-injury.

**Table 1:** Demographic and clinical characteristics of the TBI patients (mean±SD).

	TBI without sleep disturbance (n=61)	TBI with insomnia (n=28)	TBI with hypersomnia (n=9)	Total TBI (n=98)	P*
Age (years)	46±13	47±15	48±11	47±13	0.90
Male ^#^	44 (72)	19 (68)	9 (100)	72 (73)	0.16
Education (years)	11±4	9±5	9±2	10±4	0.01
Time since injury (months)	33±12	32±10	30±12	33±11	0.72
Cause of Injury ^#^					0.83
vehicle accident	40 (66)	18 (64)	5 (56)	63 (64)	
fall	16 (26)	7 (25)	3 (33)	26 (27)	
assault	3 (5)	1 (4)	0 (0)	4 (4)	
other injury	2 (3)	2 (7)	1 (11)	5 (5)	
Intracerebral Injury ^#^	35 (57)	18 (64)	8 (89)	61 (62)	0.19
GCS (n,%)					<0.01
13-15	48 (79)	18 (64)	3 (33)	69 (70)	
9-12	7 (11)	8 (29)	0 (0)	15 (15)	
3-8	6 (10)	2 (7)	6 (67)	14 (15)	
Loss of consciousness ^#^					0.50
Yes	33 (54)	19 (68)	7 (78)	59 (60)	
No	26 (43)	7 (25)	0 (0)	33 (34)	
Unknown	2 (3)	2 (7)	2 (22)	6 (6)	
Marital status ^#^					0.09
married	53 (87)	19 (68)	9 (100)	81 (83)	
single	8 (13)	9 (32)	0 (0)	17 (17)	
Headache and/or Dizziness	7 (11)	16 (57)	2 (22)	25 (26)	<0.01

Kruskal-Wallis tests were performed. Comparisons between TBI without sleep disturbance vs. TBI with insomnia, TBI without sleep disturbance vs. TBI with hypersomnia, and TBI without insomnia vs. TBI with hypersomnia were not performed for all variables.

* variation among three group medians. # n, %.

### Frequency of sleep disturbance following traumatic brain injury

**Figure 1:**
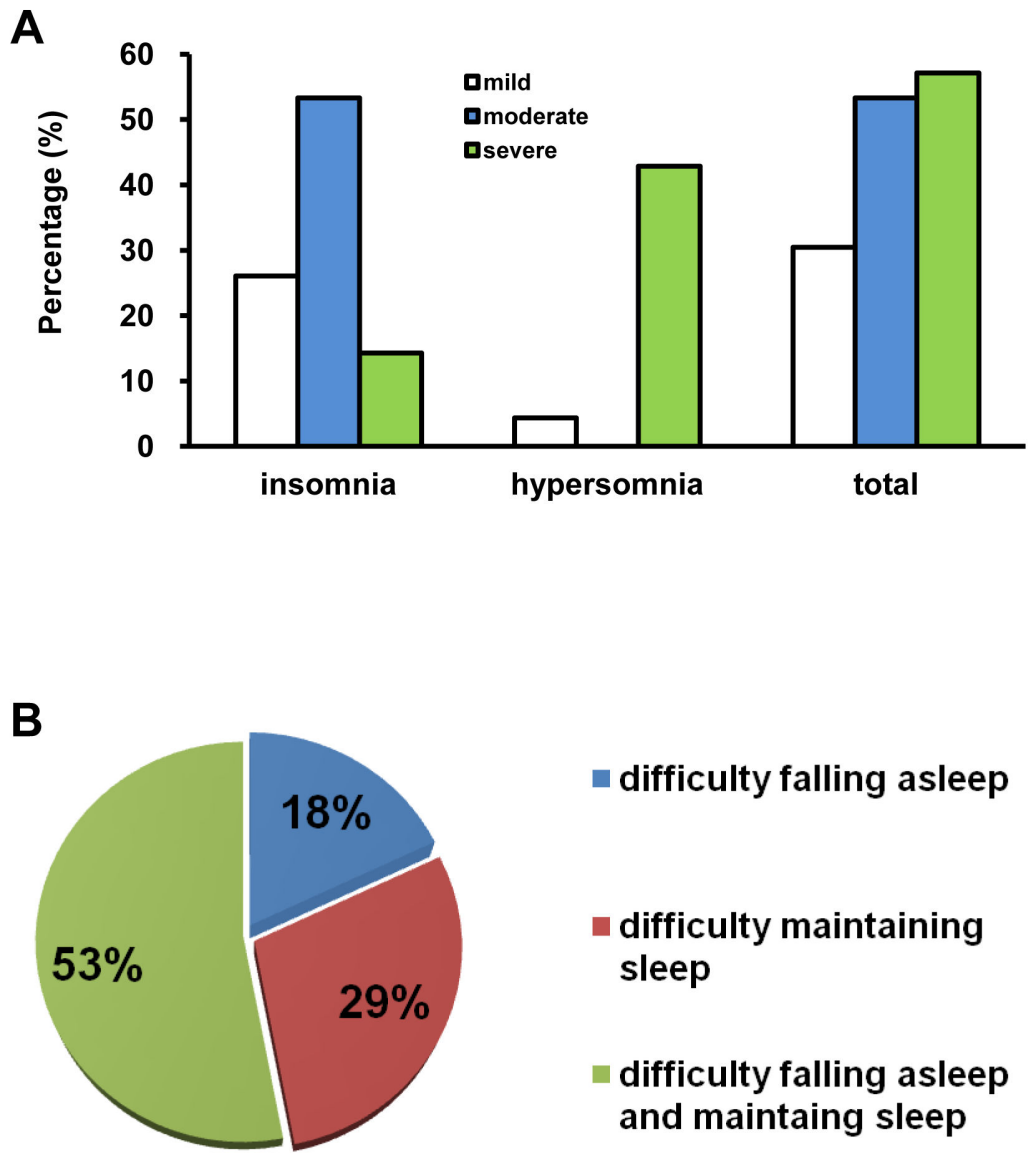
An overview on sleep disturbance in TBI population. (A) sleep disturbance in various categories of severity of TBI. (B) distribution of insomnia symptoms in TBI patients with insomnia.

37 of 98 patients (38%) reported sleep disturbance following TBI. Insomnia was diagnosed in 28 patients (29%) and post-traumatic hypersomnia in 9 patients (9%). Figure 1 offered an overview on sleep disturbance in various categories of severity of TBI. The most prevalent insomnia was found in moderate TBI (53%), while severe TBI patients were likely to experience post-traumatic hypersomnia. In total, the frequency of sleep disturbance following TBI was relatively higher in moderate (53%) and severe TBI (57%) compared to mild TBI (30%). Owing to small sample sizes in moderate and severe TBI groups, we combined these two groups into one group moderate-severe TBI group. Statistical significance for the frequency of insomnia was not seen between mild and moderate-severe TBI groups (p=0.55, χ^2^ test). In contrast, the frequency of hypersomnia was significantly different between mild and moderate-severe TBI groups (p=0.03, χ^2^ test). Totally, the frequency of sleep disturbance is statistically higher in moderate-severe TBI group compared to mild TBI group (p=0.04, χ^2^ test). In TBI with insomnia group, 5 patients (18%) complained difficulty falling asleep only, 8 patients (29%) had difficulty maintaining sleep without difficulty in initial sleep and 15 patients (53%) presented both difficulty falling asleep and difficulty maintaining sleep (Figure 1).

### Self- reported sleep quality, daytime sleepiness and psychological characteristics

**Figure 2:**
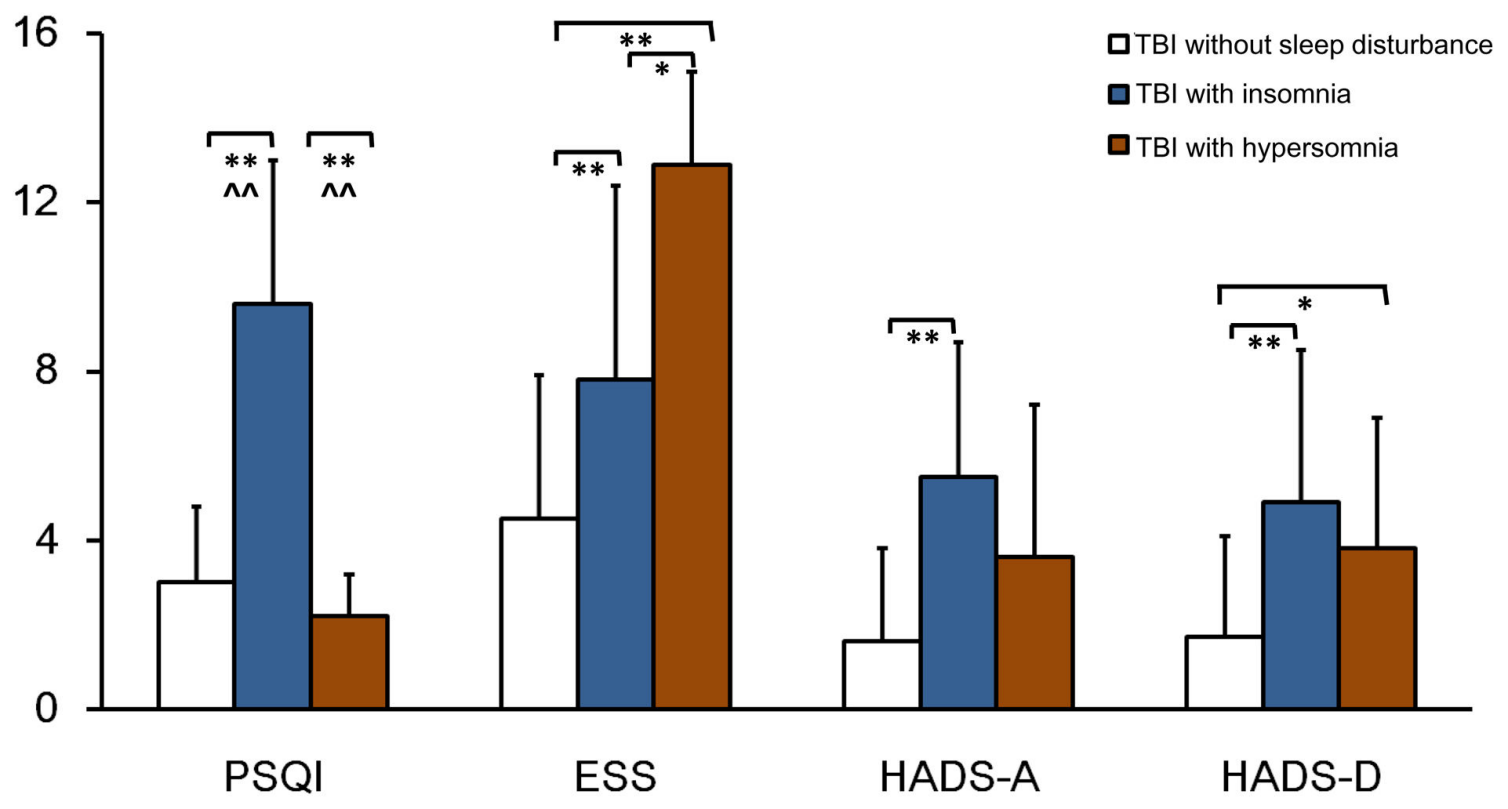
Summary of self-reported sleep quality, and psychopathology measures among TBI without sleep disturbance, TBI with insomnia and TBI with hypersomnia groups. Error bars represent mean ± SD. Significance: * p<0.05, ** p<0.01, ^ ^ p<0.01 after adjusting for HADS anxiety and depression scores.

As shown in Figure 2, TBI with insomnia group reported higher PSQI scores than TBI without sleep disturbance group (9.6±3.4 for TBI with insomnia group, 3.0±1.8 for TBI without sleep disturbance group, *p*<0.01), indicating poorer sleep quality. In contrast, the mean PSQI score of TBI with hypersomnia group was 2.2±1.0, which was not significantly different compared to TBI without sleep disturbance group. The hospital anxiety and depression scale (HADS) is a self-assessment screening questionnaire. The scoring provides information on the potential presence as well as the severity of anxiety and/or depression disorders. The HADS is now divided into four ranges: normal (0-7), mild (8-10), moderate (11-15) and severe (16-21) [25]. The average HADS scores were in the normal range in TBI without sleep disturbance and TBI with hypersomnia groups (3.15±4.26 for TBI without sleep disturbance group, 7.33±6.40 for TBI with hypersomnia group), while the mean of HADS in TBI with insomnia group was 10.39±6.25. Furthermore, HADS anxiety and HADS depression were analyzed separately. The mean scores of HADS anxiety and HADS depression in TBI with insomnia group were 5.5±3.2 and 4.9±3.6 respectively (*p*<0.01 vs. TBI without sleep disturbance group), suggesting more symptoms on anxiety and depression in TBI with insomnia patients. Similarly, a statistical difference was shown in the score of HADS depression between TBI with hypersomnia group and TBI without sleep disturbance group (3.8±3.1 vs. 1.7±2.4, p<0.05). Although average scores of HADS anxiety were higher in the TBI with hypersomnia group, the difference was not significant compared to TBI without sleep disturbance group. After adjusting for HADS anxiety and depression scores (analysis of covariance), TBI patients with insomnia were still found to have elevated PSQI scores (p<0.01). The highest ESS scores were observed in TBI with hypersomnia group. Compared to TBI without sleep disturbance group, the average ESS scores were significantly increased in the other two groups Moreover, patients with hypersomnia reported higher ESS scores than those with insomnia (12.9±2.2 for TBI with hypersomnia group, 7.8±4.6 for TBI with insomnia group, *p*<0.05).

### Identification of risk factors for sleep disturbance following TBI

Table 1 showed the results of the univariate analyses for sleep disturbance following TBI. In brief, education, severity of TBI (as measured by GCS), headache and/or dizziness were associated with sleep disturbance following TBI. Furthermore, scores of HADS anxiety and depression were significantly different among groups. Some demographic parameters, such as age, gender, time since injury and marital status were not associated with sleep disturbance following TBI in this univariate analyses. The GCS is a widely used scale for assessing level of consciousness after TBI. Compared to GCS, LOC is relatively ambiguous to describe the level of consciousness and severity of TBI. Hence we evaluated the severity of TBI with GCS rather than LOC in the present study. LOC is frequently related to deficient memory of trauma, which is generally assumed to be associated with less likelihood to develop psychiatric complications [26,27]. In our research, we considered LOC as one of potential risk factors for the development of sleep disturbance after TBI. Neither cause of injury nor LOC could influence the prevalence of sleep disturbance following TBI. Lesions on cranial CT scans were observed in frontal (n=48, 49%), temporal (n=55, 56%), parietal (n=22, 22%), occipital (n=11, 11%) lobes, diencephalon and brainstem (n=5, 5%) locations. Intracerebral injury was found in 61 (62%) patients, which is defined as the presence of intracerebral hemorrhage or contusion on CT scans. There was no significant association between location of brain injury and the presence of sleep disturbance (p=0.876). In spite of a relatively higher rate of intracerebral injury in TBI with hypersomnia group, no statistical difference was seen among three groups.

Based on the data obtained from univariate analyses, we considered education, GCS, scores of HADS anxiety, scores of HADS depression, headache and/or dizziness as potential risk factors for sleep disturbance following TBI and performed a logistic regression (Table 2). Multivariate analyses demonstrated that the variables independently associated with the presence of insomnia following TBI were score of HADS anxiety and headache and /or dizziness, and GCS was independently associated with the prevalence of hypersomnia following TBI.

**Table 2:** Multivariate Associations With Sleep Disturbance following TBI.

Variable	Insomnia		Hypersomnia	
	OR (95% CI)	*P*	OR (95% CI)	*P*
**GCS**	0.98 (0.79-1.22)	0.86	0.70 (0.55-0.90)	<0.01
**HADS-A**	1.44 (1.07-1.95)	0.02	1.02 (0.68-1. 53)	0.93
**HADS-D**	1.06 (0.79-1.41)	0.70	1.13 (0.77-1.64)	0.53
**Headache and/or dizziness**	0.10 (0.03-0.41)	<0.01	0.17 (0.02-1.48)	0.11
**Education**	0.88 (0.76-1.02)	0.09	0.83 (0.66-1.04)	0.11

## Discussion

In this cohort of observational study, we found that the overall incidence of sleep disturbance following traumatic brain injury was 38%, whereas the prevalence of insomnia is over 2 times more than that of hypersomnia. Our findings are in agreements with previous studies demonstrating that a quantitative proportion of TBI patients might experience disruption of nocturnal sleep following traumatic brain injury [5-7]. However, Baumann et al reported that EDS and/or fatigue, and hypersomnia were common after traumatic brain injury. In contrast, insomnia was extremely uncommon [14]. In their study, 37% of patients were diagnosed as severe TBI, while the majority of patients suffered mild TBI in our study. We argued that the different constitutions of patients might attribute the heterogeneities.

TBI patients with insomnia reported more symptoms of anxiety and depression, consistent with prior studies [6,7,11,28-30]. Importantly, the anxiety and depression measurement tools (HADS) do not consist of items on sleep, minimizing the confounding effect of disturbed sleep on mood measures. When the potential effect of anxiety and depression symptoms were statistically controlled, the self-reported sleep quality maintained lower in TBI patients with insomnia, suggesting that some factors beyond mood disturbance might attribute to the subjective experience of insomnia following TBI. Of 98 patients, 25 patients presented headache and/or dizziness, whereas more than half of the TBI patients with headache and/or dizziness were diagnosed as insomnia. In the study reported by Chaput et al., a high prevalence of headache and dizziness was found and the concomitant headache is strongly associated with the complaint of poor sleep in the first six weeks postinjury [31]. In the early period after TBI, some symptoms such as headache and dizziness might be temporary and gradually vanish with time [32]. In the present study, TBI patients remained headache and/or dizziness in the long-term follow up (an average of 33 months since injury), and a strong association was observed between headache and/or dizziness and the presence of insomnia. In the rehabilitation process, somatic symptoms such as headache and/or dizziness and symptoms of anxiety and depression may draw little attention from patients, family and rehabilitation professionals in China, which might be the risks factors for the presence of insomnia. On the other hand, insomnia can exacerbate these somatic and affective symptoms. All these problems can actually impede the restorative processes and reduce patients and their family’s quality of life. As for hypersomnia, we found that patients and their family didn’t perceive it to be problematic relative to other disabilities. Of note, the majority of TBI with hypersomnia suffered from severe TBI. Individuals with more severe TBI may underreport their sleep disturbance [18], probably due to their impaired self-awareness. Compared to TBI patients with insomnia, TBI patients with hypersomnia represented less somatic symptoms such as headache and/or dizziness and fewer symptoms of anxiety and depression. Although TBI patients with hypersomnia generally reported good sleep quality, they experienced more severe EDS, which might significantly affect quality of life and social functioning. Hence, more clinical attention should be paid to those TBI patients complaining of headache and/or dizziness and symptoms of anxiety and depression. Furthermore, hypersomnia and EDS should be taken seriously as well.

The findings concerning the contribution of milder TBI to sleep disturbance in TBI population have been paradoxically reported. Some data support the concept that milder TBI is associated with increased self-reported sleep disturbance. Among these studies, some drew their conclusions based on ambiguous classification of severity of TBI [6], while other tried to investigate the associations between severity of TBI and presence of sleep disturbance in the early period postinjury [11,15]. Nevertheless, recent objective sleep measures failed to confirm the relationship between milder TBI and sleep disturbance [8,17]. In contrast, more severe TBI might be linked to increased disturbed sleep [14,16,18]. Similarly, our findings failed to demonstrate a trend for patients with milder TBI to self-report poorer sleep quality. However, we did find an association between severe TBI and the development of hypersomnia in accordance with the study of Baumann et al. [14]. They found some potential causes of hypersomnia such as obstructive sleep apnea, periodic limb movements at sleep, behaviorally induced insufficiency sleep syndrome, narcolepsy without cataplexy and depression. However, other causes for hypersomnia than TBI itself could not be identified in majority of TBI patients with hypersomnia.

Aetiology and pathophysiology of sleep disturbance following TBI remain unknown. Despite the widely accepted hypothesis that damage to the cerebral structures regulating sleep might result in sleep disturbance in TBI patients, no convincing neuroimaging evidence has been obtained. Some studies found no structural abnormalities on cerebral imaging (CT or MRI scans) despite significant sleep disturbance [33,34]. Others failed to illustrate relationship between location of injury on CT scan and presence of sleep disturbance [14]. Likewise, an association between the location of brain injury and the presence of insomnia and hypersomnia wasn’t established in our sample. However, several anatomical and biochemical studies confirmed the assumption. Series of studies from Baumann et al. revealed reduced number of hypothalamic hypocretin (orexin) neurons in patients who died of severe TBI, low and undetectable cerebrospinal fluid levels of hypocretin (orexin) in the acute period after TBI and an association between low hypocretion levels and posttraumatic sleepiness [14,35,36]. Apart from hypocretin (orexin), reduced evening melatonin production due to traumatic brain damage may be associated with disturbed sleep in TBI population [18]. TBI can cause a variety of brain injuries. Some injuries, such as diffuse axonal injury, can be missed by conventional imaging [37,38] and some subtle non-structural damages also occur which can only be assessed by tissue biochemistry [39]. A lack of studies regarding precise and sensitive neuroimaging, especially metabolic and functional MRI, following TBI hinders to understand the potential mechanisms involved in the development of sleep disturbance in TBI patients.

The study has several limitations which must be taken into consideration. First, this study cohort doesn’t represent the complete TBI population. We recruited the participants with solid brain lesions observed on CT scans, whereas milder TBI patients who underwent loss of consciousness and showed no intracranial abnormalities on CT scans were not included. These milder TBI patients are prone to suffer from comorbid psychiatric disorders, which are risk factors for sleep disturbance. Second, we merely applied subjective measures for the present follow-up study, without sleep laboratory tests in our patients. It is reported that TBI patients with insomnia have a tendency to overestimate their sleep disturbance compared to polysomnographic measures of sleep [40]. Hence, the prevalence of sleep disturbance may be overvalued in the present study. Third, the size of each comparison group, especially TBI with insomnia and TBI with hypersomnia groups, was relatively small. Therefore, the findings should be interpreted and extend to general TBI patients with caution. Finally, some patients reported history of snoring. However, obstructive sleep apnea, the most common sleep disordered breathing, cannot be diagnosed by subjective measures, which might contribute to the sleep disturbance following TBI [19].

In summary, data of our well-described TBI population demonstrate that insomnia is a common and chronic condition remaining untreated in almost 30% of participants. Risk factors associated with insomnia are headache and/or dizziness and more symptoms of anxiety and depression rather than severity of TBI. In contrast, severity of TBI (assessed by GCS) is significantly associated with the presence of hypersomnia following TBI. Furthermore, there is no evidence of an association between locations of brain injury and the presence of sleep disturbance after TBI. In light of the observation that disturbed sleep persists for a long time after TBI, more clinical care and effective interventions should be taken into account.
